# TCMB: cross-model multi-level cross-attention network with Taylor-based loss for multimodal fake news detection

**DOI:** 10.3389/fdata.2026.1796969

**Published:** 2026-06-05

**Authors:** Santosh Kumar Banbhrani

**Affiliations:** 1 School of Electrical Engineering, Shaoyang University, Shaoyang, Hunan, China; 2 Department of Information and Computing, University of Sufism and Modern Science, Bhitshah, Matiari, Sindh, Pakistan

**Keywords:** deep learning, multimodal cross-attention network, multimodal fake news, social media, Taylor-based cross entropy mean bias

## Abstract

**Introduction:**

The rapid spread of misinformation across social media platforms, websites, and online communication channels has made fake news detection a critical task in the digital era. Although various computational approaches have been developed to identify fake news, many existing methods suffer from limitations such as biased training datasets and high rates of false positives and false negatives. To address these challenges, this study proposes a Multimodal Cross Attention Network with Taylor-based Cross Entropy Mean Bias (MMCN_TCMB) model for detecting multimodal fake news.

**Methods:**

The proposed approach utilizes multimodal inputs consisting of textual and visual content obtained from fake news datasets. The textual information in news posts is first tokenized using Bidirectional Encoder Representations from Transformers (BERT). Feature extraction is then performed using Word2Vec and Term Frequency–Inverse Gravity Moment (TF-IGM). Simultaneously, images associated with news posts undergo preprocessing through Contrast Limited Adaptive Histogram Equalization and Histogram Equalization (CLAHE-HE), followed by feature extraction using ResNet. The extracted textual and visual features are combined and processed through the MMCN framework. The learning mechanism of the network is enhanced using the Taylor-based Cross Entropy Mean Bias (TCMB) loss function to improve classification performance.

**Results:**

Experimental results demonstrate that the proposed MMCN_TCMB model achieves superior performance in multimodal fake news detection. The model attains a recall of 97.988%, precision of 96.223%, F1-score of 97.098%, and overall accuracy of 97.436%, outperforming existing methods.

**Discussion:**

The findings indicate that integrating multimodal feature extraction with cross-attention mechanisms and the TCMB loss function significantly enhances the reliability and accuracy of fake news detection. The proposed framework effectively captures both textual and visual inconsistencies, making it a promising approach for combating misinformation in modern digital platforms.

**The code is available on:** https://github.com/banbhrani84/MMCN_TCMB-Fake-News-.

## 1 Introduction

In recent years, platforms such as Twitter, Facebook, and Instagram have surged in popularity due to their ability to rapidly disseminate information (Kaliyar et al., 2021). As digital interactions through social media platforms become increasingly central to daily life, a growing number of individuals are turning to these platforms for news, rather than relying on traditional news outlets (Shu et al., 2017). The rapid increase in user engagement with online news is largely attributed to the inherent features of social media, such as ease of access, low cost, and the swift dissemination of information. However, despite these benefits, social media's news quality is often regarded as inferior to that of traditional news outlets. A major concern is the widespread circulation of fake news, low-quality news deliberately containing false or misleading information (Shu et al., 2018). Due to several key features of online social networks, such as ease of use, rapid information transmission, and low cost, they have become a dominant means of information and communication sharing. Today, nearly all social media users access news through online channels. However, the growing popularity of online social networks has also made the internet an ideal platform for the spread of fake news (Sahoo and Gupta, 2021). The rapid growth of these news platforms has enabled the swift and widespread distribution of fake news (Almarashy et al., 2023). Fake news manifests in diverse formats, such as misleading content, fabricated reviews, unfounded rumors, manipulative advertisements, false political rhetoric, and satirical pieces. Currently, social media platforms serve as a more efficient medium for their dissemination than mainstream media (Sahoo and Gupta, 2021).

The prevalence of unauthentic data on social media platforms has attracted significant attention from researchers, as these platforms have become key hubs for the dissemination of fake news (Tschiatschek et al., 2018). Fake news is typically written with the deliberate intent to misinform readers and tarnish the reputation of individuals, organizations, or institutions, often in pursuit of political or financial objectives (Kaliyar et al., 2021). The propagation of inaccurate claims has remained a prominent issue, particularly since the U.S. presidential election. Social media sites and online networks have come under scrutiny for their inability to effectively control the circulation of fake news. The motivations behind creating and spreading such content include political advantage, damaging corporate reputations, attracting clicks to boost ad revenue, and gaining personal attention (Tschiatschek et al., 2018). Certain types of content disseminated on social media can mislead users, evoke strong emotional responses, and erode trust. The detection and identification of fake news on these platforms pose significant challenges. Its rapid spread has the potential to impact millions and disrupt users' understanding of real-world events (Sahoo and Gupta, 2021). Detecting fake news is commonly approached as a classification problem in artificial intelligence, wherein algorithms attempt to differentiate between real and fake news articles. Despite numerous efforts to improve classification accuracy, the development of highly effective models is still an ongoing area of research (Almarashy et al., 2023). In recent years, artificial intelligence has played a transformative role across multiple disciplines, including information technology, intelligent transportation systems, virtual personal assistants, robotic surgery, and, most prominently, Natural Language Processing (NLP) (Demilie and Salau, 2022).

The detection of fake news represents a relatively new challenge that researchers are beginning to tackle (Almarashy et al., 2023). To combat the spread of fake news and its harmful impacts, many strategies have been devised (Al-Tai et al., 2023). Extensive research has been conducted on machine learning (ML)-based automatic detection techniques, which support users in assessing the veracity of news content and discerning genuine reports from particular news (Almarashy et al., 2023). Recent advancements in deep learning (DL) have established it as an emerging and powerful technology within the research community. DL offers several advantages over ML, including automated feature extraction, reduced reliance on extensive data pre-processing, the ability to capture high-dimensional features, and improved accuracy (Mridha et al., 2021). Building on the success of DL across various domains, DL-based fake news detection methods have recently been proposed and have garnered significant attention (Hua et al., 2022). DL methods, in particular, have shown superior performance over conventional ML techniques in detecting fake news (Cui et al., 2025a). As a cutting-edge technology, DL has become widely embraced in research circles, largely because of the availability of programming frameworks such as Keras and TensorFlow. Convolutional Neural Networks (CNNs) have delivered remarkable accuracy in image and text classification, whereas Recurrent Neural Networks (RNNs) have proven effective in processing sequential data, including text and speech (Al-Tai et al., 2023). Modern multimodal approaches that merge text, images, and video data demonstrate improved accuracy in detecting fake news (Qiao et al., 2025; Cui et al., 2025b, 2026). Techniques for multimodal fake news detection involve integrating and processing information from diverse media sources, which helps uncover and exploit the connections between various modalities (Li et al., 2025).

The primary research contributions are summarized below:

❖ A novel multimodal fake news detection model, MMCN_TCMB, is proposed by integrating an MMCN with a TCMB loss function to improve the classification performance and learning efficiency. The TCMB is developed by merging the Taylor concept, sigmoid cross-entropy loss, and Mean Bias Error (MBE).
❖ An effective multimodal feature extraction framework is developed by combining BERT, Word2Vec, and TF-IGM for textual feature extraction and CLAHE-HE with ResNet for visual feature extraction.
❖ An improved loss function, TCMB, is introduced to enhance the convergence performance and reduce prediction bias, resulting in improved classification accuracy and stability compared to conventional loss functions.
❖ The proposed MMCN_TCMB achieves superior performance on the Weibo and Twitter datasets, attaining a recall of 97.988%, precision of 96.223%, F1-score of 97.098%, and accuracy of 97.436%.

The remaining sections of this research are arranged as follows: Section 2 describes the available approaches for detecting fake news. Section 3 introduces the established MMCN_TCMB model along with its architectural details. Section 4 focuses on analyzing the results and discussing their significance. The final section concludes the article and identifies areas for future research.

## 2 Motivation

A detailed evaluation of recent fake news detection methodologies is presented in this section. Although numerous techniques have been developed for deepfake detection, they often fail when applied to previous multimodal inputs. Besides, modern deep learning techniques are implemented to generate fake news, complicating detection, especially due to environmental factors such as compression artifacts, changes in lighting, and extensions in scale and position. To manage these challenges efficiently, a reliable fake news detection model named MMCN_TCMB is proposed.

### 2.1 Literature review

Guo et al. (2025) proposed a Cross-modal Adaptive-aware learning for Multimodal Fake News Detection (CAMFND). This technique was more flexible, effective, and minimized the loss function. Nevertheless, this method proved inadequate for real-time applications. A Hierarchical Cross-Modal Interaction Network (HCMIN) was designed by Li et al. (2025) for detecting multimodal fake news. This model had effectively minimized overfitting and attained high generalization ability. However, the model lacked emphasis on enriching multimodal interactions, which are essential for enhancing interpretability and pinpointing the content and features driving fake news classification. Cao et al. (2025) developed Multimodal Feature Fusion Fake News Detection with Co-Attention Block (MFFFND-Co). This method had enhanced detection accuracy and prevented overfitting issues. Meanwhile, this approach did not successfully generalize feature extraction across multiple scales and cross-modal ambiguity assessment mechanisms from MFFFND-Co to accommodate a broader range of modalities. A Cross-Modal Fake News Detection Method (CM-MLF) was established by He et al. (2025). This model had high robustness and reduced the amount of computation and storage. Nonetheless, this approach failed to extend its framework to accommodate multidimensional inputs such as user interactions, sentiment cues, and comment-based features.

A Cross-modal Content Correlation Network (C3N) was introduced by Qiao et al. (2025) for improving multimodal fake news detection. This approach effectively minimized redundant information and was suitable for real-time applications. Despite its foundational strengths, the method lacked integration of supplementary information such as verified facts and user feedback, limiting its capacity for robust fake news identification. Tschiatschek et al. (2018) designed Disentanglement-based Cross-modal Clues Mining and Aggregation Network (DCCMA-Net) for explainable multimodal fake news detection. This model had faster convergence during training, reduced computational costs, and attained high generalization ability. Nevertheless, the model did not leverage the synergy between cross-modal clue extraction together with natural language generation capabilities of Language (and Vision) Language Models (L(V)LMs) to produce interpretable, reader-friendly interpretations of multimodal fake news detection. A Multi-modal Robustness Fake News Detection with Cross-Modal and Propagation Network Contrastive Learning (MFCL) was devised by Chen et al. (2025). This technique was more accurate and minimized the number of parameters required. However, this technique was not extended to heterogeneous settings, where the propagation network was structured as a heterogeneous graph. Shan et al. (2024) established Message Aggregation and a Gated Fusion network (MAGF) for fake news detection. This model had reduced computational cost and computational time. Despite its strengths, the model lacked empirical validation across diverse datasets, raising concerns about its stability and applicability in varied contexts. Wang et al. (2022) developed a Fine-grained Multimodal Fusion Network (FMFN) to detect fake news by integrating visual and textual features. In this approach, feature vectors and word embeddings were fused using a scaled dot-product attention mechanism to enhance multimodal representation. Although the method achieved improved performance in fake news detection, it did not consider social context features, which limits its ability to capture complete semantic information and affects the overall detection performance. Jing et al. (2023) proposed a Multimodal Progressive Fusion Network (MPFN) for fake news detection. Using this method, a transformer-based architecture was employed for capturing visual features at multiple levels and progressively concatenating them with textual features for improved representation. The model demonstrated good detection performance; however, it was not evaluated on deceptive or misleading content, which limits its generalization capability.

The summary of available multimodal fake news detection methods, along with their corresponding datasets, advantages, and limitations, is presented in Table 1.

**Table 1:** Summary of existing multimodal fake news detection methods.

Reference	Methodology	Dataset	Advantages	Limitations
Guo et al. (2025)	CAMFND	English Twitter and Chinese Weibo datasets	Flexible, effective, and minimized loss	Cannot be used in real-time scenarios
Li et al. (2025)	HCMIN	Twitter, Weibo, and FakeNewsNet Datasets	Reduced overfitting and good generalization	Limited multimodal interaction and interpretability
Cao et al. (2025)	MFFFND-Co	Twitter and Weibo dataset	Improved accuracy and prevented overfitting	Limited scalability to diverse modalities
He et al. (2025)	CM-MLF	Weibo and Twitter datasets	High robustness and reduced computation and storage	Did not consider multidimensional inputs such as user interactions
Qiao et al. (2025)	C3N	Weibo and Twitter datasets	Reduced redundant information and real-time capability	Did not integrate verified facts and user feedback
Tschiatschek et al. (2018)	DCCMA-Net	Facebook dataset	Fast convergence and low computational cost	Limited explainability using advanced language models
Chen et al. (2025)	MFCL	Weibo and Twitter datasets	High accuracy and reduced parameters	Not suitable for heterogeneous propagation networks
Shan et al. (2024)	MAGF	Weibo and Twitter datasets	Reduced computational cost and time	Limited validation on diverse datasets
Wang et al. (2022)	FMFN	Weibo dataset	Effective multimodal fusion using attention	Did not consider social context information
Jing et al. (2023)	MPFN	Weibo and Twitter dataset	Effective progressive multimodal fusion	Limited evaluation on deceptive and large-scale datasets

## 3 Proposed multimodal cross-attention network based on Taylor-based cross entropy mean bias for detecting fake news

**Figure 1:**
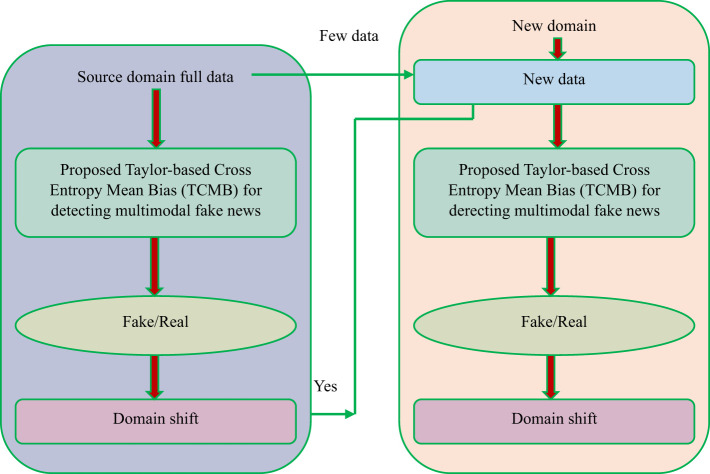
Block diagram of the MMCN_TCMB for cross-model-based multimodal fake news detection.

This study introduces an advanced model named MMCN_TCMB for detecting multimodal fake news. Initially, text and image data are collected from Weibo and Twitter. Textual data are subjected to BERT (Subakti et al., 2022)-based tokenization. Then, feature extraction is accomplished using Word2Vec (Onan, 2022) and TF-IGM (Christian et al., 2021). At the same time, the image on the news post is considered, and the image is first pre-processed via CLAHE-HE (Saifullah and Drezewski, 2023) hybrid denoising to eliminate noise and irrelevant details. Next, feature extraction is performed by utilizing ResNet (Ying et al., 2021). The extracted features from both modalities are fused and sent as input to MMCN (Ying et al., 2021). The learning strategy of MMCN is refined using the TCMB loss function. Moreover, TCMB is developed by merging the Taylor concept (Manaf et al., 2023), cross-entropy loss (Mao et al., 2023), and MBE (Jadon et al., 2024). Besides, the proposed framework addresses domain shift by checking the divergence between the source and target domains to identify the changes in data distribution. If a domain shift is identified, then the new data are combined with a few samples from the source domain data, and then it is applied to TCMB to ensure effective fake news detection across different domains. Figure 1 represents the illustration of the MMCN_TCMB for cross-model-based multimodal fake news detection.

### 3.1 Acquisition of multimodal input

Let us assume that the text and image considered for detecting fake news are taken from the dataset and the dataset is given by


$$\begin{array}{l} K_{1}=\left\{a_{1},a_{2},\ldots ,a_{j},\ldots ,a_{k}\right\} \end{array}$$ (1)

$$\begin{array}{l} K_{2}=\left\{P_{1},P_{2},\ldots ,P_{r},\ldots ,P_{s}\right\} \end{array}$$ (2)

where, *k* refers to overall number of texts, *s* represents image count, *a*_*j*_ signifies *j*^*th*^ text data, *P*_*r*_ denotes the *r*^*th*^ image, *K*_1_ stipulates the news posts as text data, and *K*_2_ specifies new posts as image.

### 3.2 News post as text

News posts as text exclude all visual elements, such as images, and focus solely on the linguistic and semantic features of the post. Analyzing the text of a news post is crucial, as fake news often relies on misleading language, emotionally charged wording, or sensational claims to influence readers. By applying NLP techniques, researchers can extract patterns related to writing style, sentiment, keyword usage, and factual consistency to assess the credibility of the content. Text-based analysis serves as a foundational component in many detecting fake news systems, enabling models, including Long Short-Term Memory (LSTMs), Transformers, or other DL architectures, to classify whether a news post is likely to be real or fake based solely on its textual features.

#### 3.2.1 BERT tokenization

In this study, BERT is used to perform tokenization. Within this approach, the textual data *a*_*j*_ is passed as input for tokenization. BERT (Subakti et al., 2022) is a robust and efficient model capable of handling various downstream tasks. It encodes both sentence pairs and single sentences into a single, unambiguous token sequence. Here, a “sentence” may denote any continuous segment of text, while a “sequence” represents an input token sequence of BERT, which can comprise one or two sentences merged. The initial token in every sequence is a special token ([*CLS*]), and the final hidden state of the special token ([*CLS*]) is exploited as a comprehensive representation of the input sequence. When two sentences are present, they are added to a single input sequence. The classification is performed via a two-stage process based on the encoded contextual information. First, a specific token ([*SEP*]) is utilized to break down a sentence, and then the learned embedding is added to each token, for identifying the sentence it is associated with. The overall input representation is then formed by summing the segment embeddings, token embeddings, and positional embeddings. The tokenized output obtained from BERT is expressed in terms of *M*.

#### 3.2.2 Feature extraction

Feature extraction identifies and derives applicable information from raw input data, such as text, to help a model differentiate between fake and real news. In this case, the tokenized result *M* is offered as input for feature extraction. Moreover, features, such as Word2vec and TF-IGM, are extracted and explained below.

**(i) Word2vec**

Word2Vec (Onan, 2022) is a neural network-based word embedding framework composed of an input layer, a hidden layer, and an output layer. Additionally, it learns vector representations of words by estimating the possibility of a target word occurring in the context of neighboring words. Word2Vec (*d*_1_) operates through two core architectures, including Skip-Gram (SG), which aims to infer surrounding context words from a given central word, whereas Continuous Bag of Words (CBOW) seeks to predict the central word using its neighboring context. CBOW utilizes neighboring words to estimate the central word, whereas SG leverages the central word to forecast its context. CBOW works well with smaller datasets, while SG tends to perform better when trained on larger datasets. Consider a set of training words {*L*_1_, *L*_2_, …, *L*_*A*_} with length *A*, the objective of SG is defined by


arg maxθ1A∑b=1A∑−J≤l≤J,l≠0logHθ(Lb+l|Lb) (3)

where, *J* denotes the training context size, *L*_*b*_ stands for the target word at the position *b*, *L*_*b*+*l*_ signifies a context word at position *b*+*l*, *l* represents an offset relative to the target word, *H*_θ_(*L*_*b*+*l*_|*L*_*b*_) represents a neural network with a sequence of parameters denoted by θ, and *H*_θ_ stands for conditional probability.

**(ii) TF-IGM**

TF-IGM (Christian et al., 2021) is conceptually similar to the well-known TF-IDF, has the ability to distinguish among distinct classes in the detection process, and can better capture the importance of a term across a corpus. The calculation of IGM and TF values is computed by


$$\begin{array}{l} TF=\frac{Count\;of\;a\;word\;in\;a\;document}{Total\;word\;count} \end{array}$$ (4)

$$\begin{array}{l} IGM=1+\lambda \left(\frac{Count\;of\;a\;word\;in\;a\;document}{Total\;word\;count}\right) \end{array}$$ (5)

$$\begin{array}{l} TF-IGM=TF\;*\;IGM \end{array}$$ (6)

Here, λ designates a scaling factor. The TF-IGM feature is indicated as *d*_2_.

The feature vector *F*_1_ attained from text is given by


$$\begin{array}{l} F_{1}=\left\{d_{1},d_{2}\right\} \end{array}$$ (7)

### 3.3 News post as image

News post as image leverages the visual elements of the news post, such as layout, fonts, embedded pictures, or screenshots, to detect signs of misinformation. By analyzing these posts as images, DL models can identify subtle visual patterns, manipulations, or inconsistencies that might indicate fake news. This image-based perspective complements traditional text analysis, allowing for a more comprehensive and effective detection of false or misleading content in online news posts.

#### 3.3.1 Image enhancement

CLAHE-HE (Saifullah and Drezewski, 2023) is an image denoising technique used for eliminating noise from images. In image denoising, the value of a target pixel is swapped by a weighted average of surrounding reference pixels, where the weights are adapted based on the similarity between the block centered on the target pixel and those centered on the reference pixels. Here, the image *P*_*r*_ serves as CLAHE-HE's input, which is utilized for image enhancement. HE serves to equalize the histogram of an image by adjusting the contrast computed using the distribution and range of intensity levels. Here, the image histogram *B*(*r*) for the pixel intensity *r* is attained based on pixel count *f*_*r*_ with pixel intensity *r* and is articulated as,


$$\begin{array}{l} B\left(r\right)=f_{r},for\;r=0,1,2,\ldots ,\left(I-1\right) \end{array}$$ (8)

wherein *I* denotes the highest gray level range.

Considering the above Equation 8, the histogram is split into the total pixels at a given intensity of the image $f=\sum_{r=0}^{I-1}B\left(r\right)$, and the outcome is exploited to compute the probability *c*_*a*_(*r*) of a pixel *a*, and is expressed by


$$\begin{array}{l} c_{a}\left(r\right)=c\left(a=r\right)=f_{r}/f \end{array}$$ (9)

The Cumulative Distribution Function (CDF) is calculated as,


$$\begin{array}{l} CDF_{a}\left(r\right)=\overset{r}{\underset{e=0}{\sum }}c_{a}\left(a=e\right) \end{array}$$ (10)

The mapping function is given by


$$\begin{array}{l} g\left(h\right)=round\left[\frac{CDF\left(h\right)-CDF_{\min}}{f-CDF_{\min}}\right] \end{array}$$ (11)

Here, *h* represents a specific gray-level intensity value. CLAHE improves image quality by applying a low threshold to limit contrast enhancement, thereby avoiding noise amplification and minimizing edge shadow effects. The limit value gained by utilizing the clip limit is articulated by


$$\begin{array}{l} \beta =\frac{F}{f}\left[1+\frac{\alpha }{100}\left(s_{\max}-1\right)\right] \end{array}$$ (12)

wherein *S*_max_ indicates maximum gray scale intensity value, *F* refers to area size, *f* stands for 8-bit gray scale value, α represents the limit of the clip factor, and *g*(*h*) signifies the new intensity value. The enhanced image obtained from CLAHE-HE is denoted by *E*.

#### 3.3.2 Feature extraction

Feature extraction regarding image-based detection of fake news identifies and extracts important visual characteristics or patterns from an image that may help determine whether the image is real or fake. Here, the enhanced image *E* is considered as input for feature extraction, and the process is performed by utilizing ResNet (Ying et al., 2021).

**(i) ResNet**

ResNet (Ying et al., 2021) has demonstrated superior capability in capturing representative and discriminative features over the Visual Geometry Group (VGG). The feature map obtained from the penultimate pooling layer of ResNet is constructed by concatenating the region-wise features extracted from the enhanced image *E* and can be formally defined as follows,


F2={g1,g2,.....gb}=ResNet(E) (13)

Here, *F*_2_ = {*g*_1_, *g*_2_, .....*g*_*b*_} stipulates image region features' concatenation, $g_{b}\in {\mathbb{R}}^{n_{o}}$ characterizes features corresponding to a particular image area *b*, *n*_*o*_ symbolizes the dimension of the region feature, and *q* denotes several regions. The feature vector attained using ResNet is expressed as *F*_2_.

In *n*_*s*_ ≠ *n*_*o*_, a 2D-convolutional layer is added to convert image resolution area features to be similar to the dimension of textual word features, and is represented as *n*_*o*_ = *n*_*s*_ = *n*_*j*_. Here, *n*_*s*_ signifies the dimension of the textual feature vector and *n*_*j*_ refers to the shared dimension after alignment.

The resultant extracted feature *N* is obtained by combining the ResNet feature *F*_2_ and the feature vector *F*_1_ attained from text, and is given as follows,


$$\begin{array}{l} N=\left\{F_{1}||F_{2}\right\} \end{array}$$ (14)

### 3.4 Fake news detection using the proposed MMCN-TCMB

Here, the merged features *N* are provided as input and applied to MMCN (Ying et al., 2021) with the proposed TCMB, which is used for detecting fake news. Moreover, MMCN_TCMB is developed by updating the learning rule of MMCN using TCMB, where TCMB is the hybridization of the Taylor concept (Manaf et al., 2023), cross-entropy loss (Mao et al., 2023), with MBE (Jadon et al., 2024). MMCN effectively fuses text and image data, capturing inter-modal relationships, improving contextual understanding, enhancing fake news detection accuracy, and enabling robust reasoning across diverse information sources.

#### 3.4.1 Architecture of MMCN

MMCN is a network used to fuse text and image features using attention mechanisms to represent relationships both within and between modalities, enhancing fake news detection through rich, aligned semantic representations across modalities. In MMCN, the concatenation of both text and image data, $N=\left(\begin{array}{c} F_{1}\\ F_{2} \end{array}\right)=\left\{d_{1};.....,d_{a};g_{1};.....,g_{b}\right\}$, is given as input; here $N\in {\mathbb{R}}^{\left(a+b\right)\times n_{j}}$, *F*_1_ = {*d*_1_, *d*_2_, ......, *d*_*a*_}, and *F*_2_ = {*g*_1_, *g*_2_, .....*g*_*b*_}, in which image region features are improved to a similar dimension. The feature concatenation *N* is subjected to a transformer unit. The key *R*_*N*_, value *U*_*N*_, and query *T*_*N*_ for fine-grained features are articulated by


$$\begin{array}{l} R_{N}=NS^{R}=\left(\begin{array}{c} F_{2}S^{R}\\ F_{1}S^{R} \end{array}\right)=\left(\begin{array}{c} R_{F_{2}}\\ R_{F_{1}} \end{array}\right) \end{array}$$ (15)

$$\begin{array}{l} T_{N}=NS^{T}=\left(\begin{array}{c} F_{2}S^{T}\\ F_{1}S^{T} \end{array}\right)=\left(\begin{array}{c} T_{F_{2}}\\ T_{F_{1}} \end{array}\right) \end{array}$$ (16)

$$\begin{array}{l} U_{N}=NS^{U}=\left(\begin{array}{c} F_{2}S^{T}\\ F_{1}S^{T} \end{array}\right)=\left(\begin{array}{c} U_{F_{2}}\\ U_{F_{1}} \end{array}\right) \end{array}$$ (17)

where *S* denotes a learnable parameter matrix.

Then, the Scaled Dot-Product Attention is expressed by


$$\begin{array}{l} Attention\left(T_{N},R_{N},U_{N}\right)=soft\max\left(\frac{T_{N}\overset{\text{T}}{\underset{N}{R}}}{\sqrt{w}}\right)U_{N} \end{array}$$ (18)

wherein *w* refers to the scaling factor. To simplify the derivation and enhance interpretability, the softmax and scaling operations are removed in Equation 19 while preserving the core concept of the attention mechanism. Then, the revised equation is given by


$$\begin{array}{l} T_{N}R_{N}^{\text{T}}U_{N}=\left(\begin{array}{c} T_{F_{1}}\\ T_{F_{2}} \end{array}\right)\left(\begin{array}{cc} R_{F_{1}}^{\text{T}} & R_{F_{2}}^{\text{T}} \end{array}\right)\left(\begin{array}{c} U_{F_{1}}\\ U_{F_{2}} \end{array}\right) \end{array}$$ (19)

$$\begin{array}{l} T_{N}R_{N}^{\text{T}}U_{N}=\left(\begin{array}{cc} T_{F_{1}}R_{F_{1}}^{\text{T}} & T_{F_{1}}R_{F_{2}}^{\text{T}}\\ T_{F_{2}}R_{F_{1}}^{\text{T}} & T_{F_{2}}R_{F_{2}}^{\text{T}} \end{array}\right)\left(\begin{array}{c} U_{F_{1}}\\ U_{F_{2}} \end{array}\right) \end{array}$$ (20)

$$\begin{array}{l} T_{N}R_{N}^{\text{T}}U_{N}=\left(\begin{array}{c} T_{F_{1}}R_{F_{1}}^{\text{T}}U_{F_{1}}+T_{F_{1}}R_{F_{1}}^{\text{T}}U_{F_{2}}\\ T_{F_{2}}R_{F_{1}}^{\text{T}}U_{F_{2}}+T_{F_{2}}R_{F_{1}}^{\text{T}}U_{F_{1}} \end{array}\right) \end{array}$$ (21)

After the attention layer, $\left(\begin{array}{c} F_{1_{up}}\\ F_{2_{up}} \end{array}\right)=T_{N}R_{N}^{\text{T}}U_{N}$, upgraded features of image and text fragments are computed as,


$$\begin{array}{l} F_{1_{up}}=\left\{d_{up1};......;d_{upa}\right\}=T_{F_{1}}R_{F_{1}}^{\text{T}}U_{F_{1}}+T_{F_{1}}R_{F_{1}}^{\text{T}}U_{F_{2}} \end{array}$$ (22)

$$\begin{array}{l} F_{2_{up}}=\left\{g_{up1};......;g_{upb}\right\}=T_{F_{2}}R_{F_{1}}^{\text{T}}U_{F_{2}}+T_{F_{2}}R_{F_{1}}^{\text{T}}U_{F_{1}} \end{array}$$ (23)

The results demonstrate that the transformer's multi-head sublayer output reflects meaningful interactions across different modalities. Afterward, $\left(\begin{array}{c} F_{1_{up}}\\ F_{2_{up}} \end{array}\right)$is applied to the position-wise forward sublayer, and the resultant obtained is indicated as $N_{k}=\left(\begin{array}{c} F_{1_{k}}\\ F_{2_{k}} \end{array}\right)$. Then, *N*_*k*_ is split into *F*_1_*k*__ = {*d*_*k*1_, ....., *d*_*ka*_} and *F*_2_*k*__ = {*g*_*k*1_, ...., *g*_*kb*_}. By leveraging the Text_CNN module, designed to extract n-gram-like local textual features before aggregation, the model achieves superior performance. The text feature *F*_1_*k*__ is subjected to Text_CNN for aggregating and is rewritten as,


$$\begin{array}{l} F_{1_{j}}=Text\text{\_}CNN\left(F_{1_{k}}\right) \end{array}$$ (24)

The concatenation of image region features *F*_2_*k*__ is aggregated using an average pooling layer, described mathematically as follows,


$$\begin{array}{l} F_{2_{j}}=\frac{1}{b}\overset{b}{\underset{s=1}{\sum }}u_{ks} \end{array}$$ (25)

The resultant multi-modal feature representation of posts is obtained by the sum operation among *F*_2_*J*__ and *F*_1_*J*__, and is given by


$$\begin{array}{l} P=\lambda F_{1_{j}}+\left(1-\lambda \right)F_{2_{j}} \end{array}$$ (26)

wherein λ characterizes the ratio factor of text and image information in multi-modal features.

**(a) Multi-modal encoding network**

The Multi-Level Encoding Network gathers hierarchical features from data by processing inputs at multiple abstraction levels. In fake news detection, it extracts both global and local semantic cues from text or images, improving understanding and classification. This layered approach enhances model performance by preserving fine-grained details and contextual representations. The two multi-modal representations conforming to distinct semantic levels are evaluated and are represented as


$$\begin{array}{l} P^{i}=\lambda_{1}F_{1_{aj}}+\left(1-\lambda_{1}\right)F_{2_{aj}} \end{array}$$ (27)

$$\begin{array}{l} P^{j}=\lambda_{2}F_{1_{aj}}+\left(1-\lambda_{2}\right)F_{2_{aj}} \end{array}$$ (28)

Here, *P*^*i*^ signifies high-level multi-modal semantic feature and the low-level feature is represented as *P*^*j*^. The concatenated output for two units is defined by


$$\begin{array}{l} P=concate\left(P^{i},P^{j}\right) \end{array}$$ (29)

Here, *concate* refers to the concatenate operation and *P* represents the overall multi-modal representation output.

**(b) Fake news classification network**

**Figure 2:**
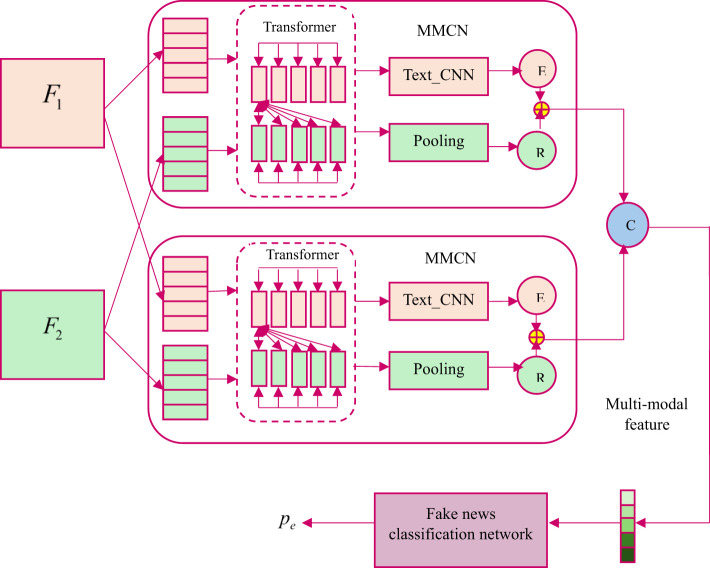
MMCN architecture.

Considering the multi-modal representation, *P* = {*l*_1_, ...., *l*_*Q*_}, fake news classification network is utilized to identify real vs. deceptive posts. It employs a fully connected layer with softmax to determine the likelihood of a post being fake, and the classification result is given by


$$\begin{array}{l} p_{e}=\sigma \left(S_{q}l_{n}+w\right) \end{array}$$ (30)

where σ(.) stands for the softmax activation function, *p*_*e*_ states the possibility that the post *n* is fake, and *l*_*n*_ indicates the post feature vector *n*. Furthermore, to characterize the ground-truth label of the post *n*, the output obtained from MMCN is represented by *p*_*e*_. The structure of MMCN is shown in Figure 2.

#### 3.4.2 Proposed multimodal cross-attention network-based Taylor-based cross-entropy mean bias

The learning rule of the MMCN is refined using TCMB, which is developed by integrating the Taylor series (Manaf et al., 2023), cross-entropy loss (Mao et al., 2023), and MBE (Jadon et al., 2024). MBE reveals systematic prediction bias, offers clear interpretability, aids model validation, and guides correction for consistent over- or underestimation. The sigmoid cross-entropy loss improves classification accuracy and supports gradient-based optimization for robust model training. Taylor's concept boosts efficiency, reduces costs, simplifies training, enhances specialization, improves control, increases productivity, and ensures better performance. Thus, TCMB improves classification accuracy by reducing bias and optimizing loss functions, leading to more reliable and context-aware fake news detection across platforms.

**Step 1: Take two loss functions**

Here, two loss functions, namely MBE (Jadon et al., 2024) and sigmoid cross entropy (Mao et al., 2023), are considered, which are utilized for updating the learning rule of MMCN detecting fake news.

**(i) Mean bias error**

MBE (Jadon et al., 2024) is a metric that helps determine the accuracy of a predictive model. It also reveals whether the model has a consistent bias, either predicting values too high or too low. The MBE (*L*_*MBE*_) is expressed by


$$\begin{array}{l} L_{MBE}=\frac{1}{X}\overset{X}{\underset{e=1}{\sum }}\left(p_{e}-{\hat{p}}_{e}\right) \end{array}$$ (31)

where *X* indicates the number of data samples, *p*_*e*_ represents the actual output of MMCN, and ${\hat{p}}_{e}$ specifies the expected value.

**(ii) Sigmoid cross entropy**

The Sigmoid cross-entropy loss function (*L*_*SCE*_), which offers interpretability and contributes to model stability, is measured by


$$\begin{array}{l} L_{SCE}=-\left[{\hat{p}}_{e}\log\left(\sigma \left(p_{e}\right)\right)+\left(1-{\hat{p}}_{e}\right)\log\left(1-\sigma \left(p_{e}\right)\right)\right] \end{array}$$ (32)

$$\begin{array}{l} \sigma \left(p_{e}\right)=\frac{1}{1-e^{-p}} \end{array}$$ (33)

wherein σ(.) signifies the sigmoid function.

**Step 2:** Use the Taylor series on the individual loss

Taylor series is given for individual loss functions to capture their behavior locally, enabling gradient analysis, simplifying optimization, and improving convergence in training via computationally efficient and interpretable modeling. Assume a second-order expansion of the Taylor series (Manaf et al., 2023) and is defined by


$$\begin{array}{l} Y\left(z\right)=Y\left(z-1\right)+Y^{\prime }\left(z-1\right)+\frac{1}{2}Y^{\prime \prime }\left(z-1\right) \end{array}$$ (34)

wherein the first-order derivative at the point *z* is exemplified as *Y*′(*z*), the function value at the point *z* is signified as *Y*(*z*), and second-order derivative at a specific point *z* is represented as *Y*″(*z*). Equation 34 is rephrased by


$$\begin{array}{l} Y\left(z\right)=Y\left(z-1\right)+\left(Y\left(z-1\right)-Y\left(z-2\right)\right)+\frac{1}{2}[Y\left(z-1\right)\\ \;-2Y\left(z-2\right)+Y\left(z-3\right)] \end{array}$$ (35)

$$\begin{array}{l} Y\left(z\right)=Y\left(z-1\right)+Y\left(z-1\right)-Y\left(z-2\right)+\frac{1}{2}Y\left(z-1\right)\\ \;-\frac{1}{2}2Y\left(z-2\right)+\frac{1}{2}Y\left(z-3\right) \end{array}$$ (36)

$$\begin{array}{l} Y\left(z\right)=\frac{5}{2}Y\left(z-1\right)-2Y\left(z-2\right)+\frac{1}{2}Y\left(z-3\right) \end{array}$$ (37)

The equation for MBE is expressed as,


$$\begin{array}{l} L_{MBE}\left(z\right)=\frac{1}{X}\overset{X}{\underset{e=1}{\sum }}\left(p_{e}\left(z\right)-{\hat{p}}_{e}\left(z\right)\right) \end{array}$$ (38)

By applying Taylor in MBE, then Equation 38 becomes,


$$\begin{array}{l} L_{MBE}\left(z\right)=\frac{5}{2}L_{MBE}\left(z-1\right)-2L_{MBE}\left(z-2\right)\\ \;+\frac{1}{2}L_{MBE}\left(z-3\right) \end{array}$$ (39)

$$\begin{array}{l} L_{MBE}\left(z\right)=\frac{5}{2}\left(\frac{1}{X}\sum_{e=1}^{X}\left(p_{e}\left(z-1\right)-{\hat{p}}_{e}\right)\right)\\ \;-2\left(\frac{1}{X}\sum_{e=1}^{X}\left(p_{e}\left(z-2\right)-{\hat{p}}_{e}\right)\right)\\ \;+\frac{1}{2}\left(\frac{1}{X}\sum_{e=1}^{X}\left(p_{e}\left(z-3\right)-{\hat{p}}_{e}\right)\right) \end{array}$$ (40)

The following expression yields the sigmoid cross-entropy loss,


$$\begin{array}{l} L_{SCE}\left(z\right)=-\left[{\hat{p}}_{e}\log\left(\sigma \left(p_{e}\left(z\right)\right)\right)+\left(1-{\hat{p}}_{e}\right)\log\left(1-\sigma \left(p_{e}\left(z\right)\right)\right)\right] \end{array}$$ (41)

When the Taylor series is applied to the sigmoid cross-entropy loss function, Equation 41 becomes,


$$\begin{array}{l} L_{SCE}\left(z\right)=\frac{5}{2}L_{SCE}\left(z-1\right)-2L_{SCE}\left(z-2\right)+\frac{1}{2}L_{SCE}\left(z-3\right) \end{array}$$ (42)

$$L_{SCE}\left(z\right)=-\left[\begin{array}{ll} \; & \frac{5}{2}[{\hat{p}}_{e}\log\left(\sigma \left(p_{e}\left(z-1\right)\right)\right)\\ \; & +\left(1-{\hat{p}}_{e}\right)\log\left(1-\sigma \left(p_{e}\left(z-1\right)\right)\right)]-\\ \; & 2[{\hat{p}}_{e}\log\left(\sigma \left(p_{e}\left(z-2\right)\right)\right)\\ \; & +\left(1-{\hat{p}}_{e}\right)\log\left(1-\sigma \left(p_{e}\left(z-2\right)\right)\right)]+\\ \; & \frac{1}{2}[{\hat{p}}_{e}\log\left(\sigma \left(p_{e}\left(z-3\right)\right)\right)\\ \; & +\left(1-{\hat{p}}_{e}\right)\log\left(1-\sigma \left(p_{e}\left(z-3\right)\right)\right)] \end{array}\right]$$ (43)

**Step 3: Hybrid loss function**

The hybrid loss function *L*_*Hyb*_(*z*) is designed by incorporating weighted MBE *L*_*MBE*_(*z*) and Sigmoid cross-entropy *L*_*SCE*_(*z*) and is defined by


$$\begin{array}{l} L_{Hyb}\left(z\right)=L_{MBE}\left(z\right)+\lambda L_{SCE}\left(z\right) \end{array}$$ (44)

where λ refers to a constant.

Substituting both loss function values in Equation 44, then


$$\begin{array}{l} L_{Hyb}\left(z\right)=\frac{5}{2}\left(\frac{1}{X}\sum_{e=1}^{X}\left(p_{e}\left(z-1\right)-{\hat{p}}_{e}\right)\right)\\ \;-2\left(\frac{1}{X}\sum_{e=1}^{X}\left(p_{e}\left(z-2\right)-{\hat{p}}_{e}\right)\right)\\ \;+\frac{1}{2}\left(\frac{1}{X}\sum_{e=1}^{X}\left(p_{e}\left(z-3\right)-{\hat{p}}_{e}\right)\right)-\\ \;\lambda \left[\begin{array}{l} \frac{5}{2}[{\hat{p}}_{e}\log\left(\sigma \left(p_{e}\left(z-1\right)\right)\right)\\ +\left(1-{\hat{p}}_{e}\right)\log\left(1-\sigma \left(p_{e}\left(z-1\right)\right)\right)]-\\ 2[{\hat{p}}_{e}\log\left(\sigma \left(p_{e}\left(z-2\right)\right)\right)\\ +\left(1-{\hat{p}}_{e}\right)\log\left(1-\sigma \left(p_{e}\left(z-2\right)\right)\right)]+\\ \frac{1}{2}[{\hat{p}}_{e}\log\left(\sigma \left(p_{e}\left(z-3\right)\right)\right)\\ +\left(1-{\hat{p}}_{e}\right)\log\left(1-\sigma \left(p_{e}\left(z-3\right)\right)\right)] \end{array}\right] \end{array}$$ (45)

**Step 4: Learning rule decomposition**

The influence of the loss function becomes more transparent by decomposing the learning rule (Gonzalez et al., 2025). This methodology facilitates the assessment of loss functions used in various steps of the training process. The conventional parameter update procedure in Stochastic Gradient Descent (SGD) is defined as


$$\begin{array}{l} \theta \leftarrow \theta -\eta \nabla_{\theta }\left(L_{Hyb}\left(N_{e},{\hat{p}}_{e},\theta \right)\right) \end{array}$$ (46)

Here, the learning rate is designated η, $L_{Hyb}\left(N_{e},{\hat{p}}_{e},\theta \right)$ stands for the loss function, the gradient of θ is indicated as ∇_θ_, the input applied to MMCN is described as *N*_*e*_. For a single weight θ_*i*_, the upgraded rule is,


$$\begin{array}{lll} \theta_{i}^{z+1} & \leftarrow & \theta_{i}^{z}-\eta D_{i}\left(L_{Hyb}\left(N_{e}^{z},{\hat{p}}_{e},\theta_{e}\right)\right) \end{array}$$ (47)

$$\begin{array}{lll} \theta_{i}^{z+1} & \leftarrow & \theta_{i}^{z}-\eta \frac{\partial }{\partial y}{\left[L_{Hyb}\left(N_{e},{\hat{p}}_{e},\theta_{D}\right)+y_{i}\right]}_{y\to 0} \end{array}$$ (48)

wherein, η denotes a bias vector. Let us assume, *p*_*e*_(*z*−1) = *p*_*e*_(*N*_*e*_, θ_*z*−1_), *p*_*e*_(*z*−3) = *p*_*e*_(*N*_*e*_, θ_*z*−3_), and *p*_*e*_(*z*−2) = *p*_*e*_(*N*_*e*_, θ_*z*−2_). Equation 47 is rewritten as,


$$\begin{array}{l} \theta_{i}^{z+1}\leftarrow \theta_{i}^{z}+\eta \frac{\partial }{\partial y}\\ {\left(\begin{array}{l} \frac{1}{X}\sum_{e=1}^{X}\left[\begin{array}{l} \frac{5}{2}\left(p_{e}\left(N_{e},\theta_{z-1}\right)\frac{\partial }{\partial y}\left(N_{e},\theta_{z-1}+y_{i}\right)\right)-\\ 2\left(p_{e}\left(N_{e},\theta_{z-2}\right)\frac{\partial }{\partial y}\left(N_{e},\theta_{z-1}+y_{i}\right)\right)+\\ \frac{1}{2}\left(p_{e}\left(N_{e},\theta_{z-3}\right)\frac{\partial }{\partial y}\left(N_{e},\theta_{z-1}+y_{i}\right)\right) \end{array}\right]\\ -\lambda \left[\begin{array}{l} \frac{5}{2}(\frac{{\hat{p}}_{e}}{\sigma \left[p_{e}\left(N_{e},\theta_{z-1}\right)\right]}\frac{\partial }{\partial y}\sigma \left[p_{e}\left(N_{e},\theta_{z-1}+y_{i}\right)\right]\\ -\frac{\left(1-{\hat{p}}_{e}\right)}{1-\sigma \left[p_{e}\left(N_{e},\theta_{z-1}\right)\right]}\frac{\partial }{\partial y}\sigma \left[p_{e}\left(N_{e},\theta_{z-1}+y_{i}\right)\right])-\\ 2(\frac{{\hat{p}}_{e}}{\sigma \left[p_{e}\left(N_{e},\theta_{z-2}\right)\right]}\frac{\partial }{\partial y}\sigma \left[p_{e}\left(N_{e},\theta_{z-2}+y_{i}\right)\right]\\ -\frac{\left(1-{\hat{p}}_{e}\right)}{1-\sigma \left[p_{e}\left(N_{e},\theta_{z-2}\right)\right]}\frac{\partial }{\partial y}\sigma \left[p_{e}\left(N_{e},\theta_{z-2}+y_{i}\right)\right])+\\ \frac{1}{2}\left(\frac{{\hat{p}}_{e}}{\sigma \left[p_{e}\left(N_{e},\theta_{z-3}\right)\right]}\frac{\partial }{\partial y}\sigma \left[p_{e}\left(N_{e},\theta_{z-3}+y_{i}\right)\right]\right)\\ -\frac{\left(1-{\hat{p}}_{e}\right)}{1-\sigma \left[p_{e}\left(N_{e},\theta_{z-3}\right)\right]}\frac{\partial }{\partial y}\sigma \left[p_{e}\left(N_{e},\theta_{z-3}+y_{i}\right)\right] \end{array}\right] \end{array}\right)}_{y\to 0} \end{array}$$ (49)

The overall resultant of TCMB is given by


$$\begin{array}{l} \theta_{i}^{z+1}\leftarrow \theta_{i}+\eta \\ \;\left(\begin{array}{l} \frac{1}{X}\sum_{e=1}^{X}\left[\begin{array}{l} \;\frac{5}{2}\left(p_{e}\left(V_{e},\theta_{z-1}\right)\right)-\\ \;3\left(p_{e}\left(V_{e},\theta_{z-2}\right)\right)+\\ \;\frac{1}{2}\left(p_{e}\left(V_{e},\theta_{z-3}\right)\right) \end{array}\right]\\ -\lambda \left[\begin{array}{l} \;\frac{5}{2}\left(\frac{{\hat{p}}_{e}}{\sigma \left[p_{e}\left(V_{e},\theta_{z-1}\right)\right]}-\frac{\left(1-{\hat{p}}_{e}\right)}{1-\sigma \left[p_{e}\left(V_{e},\theta_{z-1}\right)\right]}\right)-\\ \;2\left(\frac{{\hat{p}}_{e}}{\sigma \left[p_{e}\left(V_{e},\theta_{z-2}\right)\right]}-\frac{\left(1-{\hat{p}}_{e}\right)}{1-\sigma \left[p_{e}\left(V_{e},\theta_{z-2}\right)\right]}\right)+\\ \;\frac{1}{2}\left(\frac{{\hat{p}}_{e}}{\sigma \left[p_{e}\left(V_{e},\theta_{z-3}\right)\right]}-\frac{\left(1-{\hat{p}}_{e}\right)}{1-\sigma \left[p_{e}\left(V_{e},\theta_{z-3}\right)\right]}\right) \end{array}\right] \end{array}\right) \end{array}$$ (50)

where the data index is stated as *e*, time is indicated as *z*, and the weight index is represented as *i*. TCMB enhances robustness to label noise, improves generalization, balances bias and variance, enables flexible approximation, and unifies multiple loss functions for more stable deep model training. Its stable gradient flow supports the MMCN in learning intricate patterns efficiently, resulting in accelerated convergence and improved classification performance. The pseudo-code of the MMCN_TCMB is given in Algorithm 1.

Algorithm 1 **Pseudo–code of the MMCN_TCMB.**

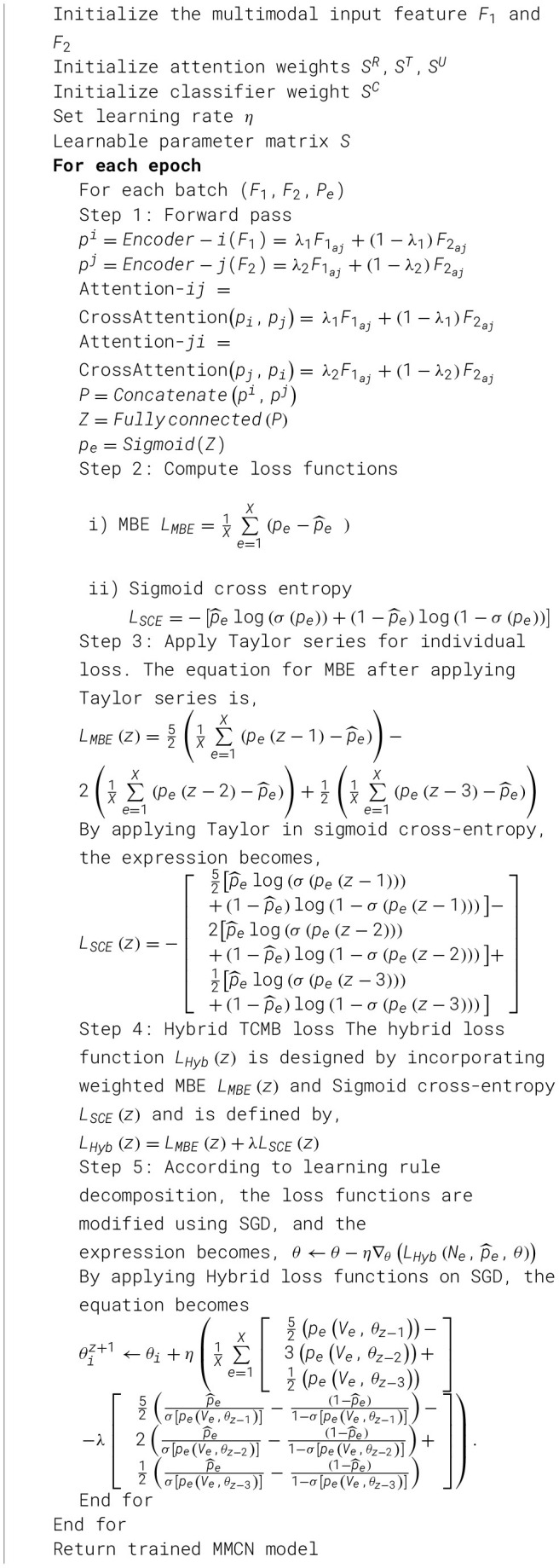

## 4 Results and discussion

The comprehensive estimation of MMCN_TCMB for detecting multimodal fake news regarding several standard metrics is presented here.

### 4.1 Experimental setup

The MMCN_TCMB for detecting multimodal fake news is executed by PYTHON tool. The network training utilized a system with an NVIDIA GTX 1080 GPU, Intel i7-6700 CPU, 16 GB RAM, and Ubuntu 16.04.

### 4.2 Performance measures

The efficacy of MMCN_TCMB in detecting fake news is assessed using performance metrics, such as precision, F1-score, and recall, which are demonstrated as follows:

**(a) Precision**

Precision appraises the accuracy of positive detections achieved by MMCN_TCMB and is formulated by


$$\begin{array}{l} \mathrm{Pr}ecision=\frac{D}{D+G} \end{array}$$ (51)

where *D* signifies true positive and *G* symbolizes a false positive.

**(b) Recall**

Recall measures the possibility that the positive instances accurately detected exploiting MMCN_TCMB are actually positive and is given by


Recall=DD+E (52)

wherein *E* indicates a false negative.

**(c) F1-score**

The F1-score is computed as the harmonic mean of recall and precision and is defined as


$$\begin{array}{l} F1-score=\frac{2\times recall\times precision}{recall+precision} \end{array}$$ (53)

**(d) Accuracy**

Accuracy is defined as the ratio of correctly classified fake and real news samples to the total number of samples, which reflects the overall effectiveness of the proposed MMCN_TCMB model.


$$\begin{array}{l} Accuracy=\frac{D+F}{D+F+G+E} \end{array}$$ (54)

wherein *F* indicates a true negative.

**(e) Receiver operating characteristics curve**

ROC provides a graphical depiction of a binary model's performance, indicating True Positive Rate (TPR) and False Positive Rate (FPR) at multiple threshold settings.

### 4.3 Dataset description

Here, two multimodal data, such as image and text, are taken from two datasets, namely Fakeddit Multimodal Fake News Classification (Dataset 1) (Kaggle, 2025) and Weibo and Twitter dataset (Dataset 2) (IEEE DataPort, 2025), for detecting fake news.

**(i) Fakeddit multimodal fake news classification dataset (dataset 1)**

This dataset (Kaggle, 2025) is a large-scale collection of Reddit posts designed to aid in detecting misinformation by leveraging both textual content and associated images. Fakeddit comprises hundreds of thousands of annotated posts designed for fake news classification, enabling the development of models that jointly process textual and visual modalities. By integrating these data types, Fakeddit offers a comprehensive and realistic dataset for examining misinformation in social media environments, establishing itself as a critical benchmark for advancing the detection of multimodal fake news research. The details of the dataset are presented in Table 2.

**Table 2:** Description of dataset-1.

File name	Description	File size (MB)
multimodal_train.tsv	Training data containing text and image information for model learning	155.66
multimodal_validate.tsv	Validation data used for tuning model parameters	16.34
multimodal_test_public.tsv	Test data used for evaluating model performance	16.40

**(ii) Weibo and Twitter dataset (dataset 2)**

This database (IEEE DataPort, 2025) is a popular resource for studying misinformation and fake news on social media platforms. The Weibo dataset comprises posts sourced from Sina Weibo, a prominent Chinese microblogging service, and is frequently annotated for tasks such as fake news detection, rumor classification, and misinformation analysis. Simultaneously, the Twitter dataset includes annotated tweets from the Twitter platform, facilitating the identification of false information, rumors, and deceptive content. The Twitter dataset originates from the Twitter platform and consists of brief textual posts, frequently supplemented with visual media such as images or videos. Each data instance integrates both a news-related text and a corresponding visual element, supporting multimodal analysis. The detailed characteristics of the datasets are presented in Table 3.

**Table 3:** Description of dataset-2.

Parameter	Description
Total size	2.4 GB
Training:validation:test split	7:1:2
Twitter rumors	~6,000
Twitter non-rumors	~5,000
Number of rumor events	11
Data instance	Text with corresponding image

### 4.4 Experimental parameters

The experimental parameters for MMCN_TCMB for detecting multimodal fake news are presented in Table 4.

**Table 4:** Experimental parameters for MMCN_TCMB.

Parameters	Values
Epoch	50
Batch size	64
Optimizer	Adam
Learning rate	0.01
beta2	0.99
Kernel size	3 × 3
beta1	0.9
Epsilon	0.0001
Loss	TMCB
Activation	Sigmoid
Dropout	0.3

### 4.5 Explainability results using GradCAM

**Figure 3:**
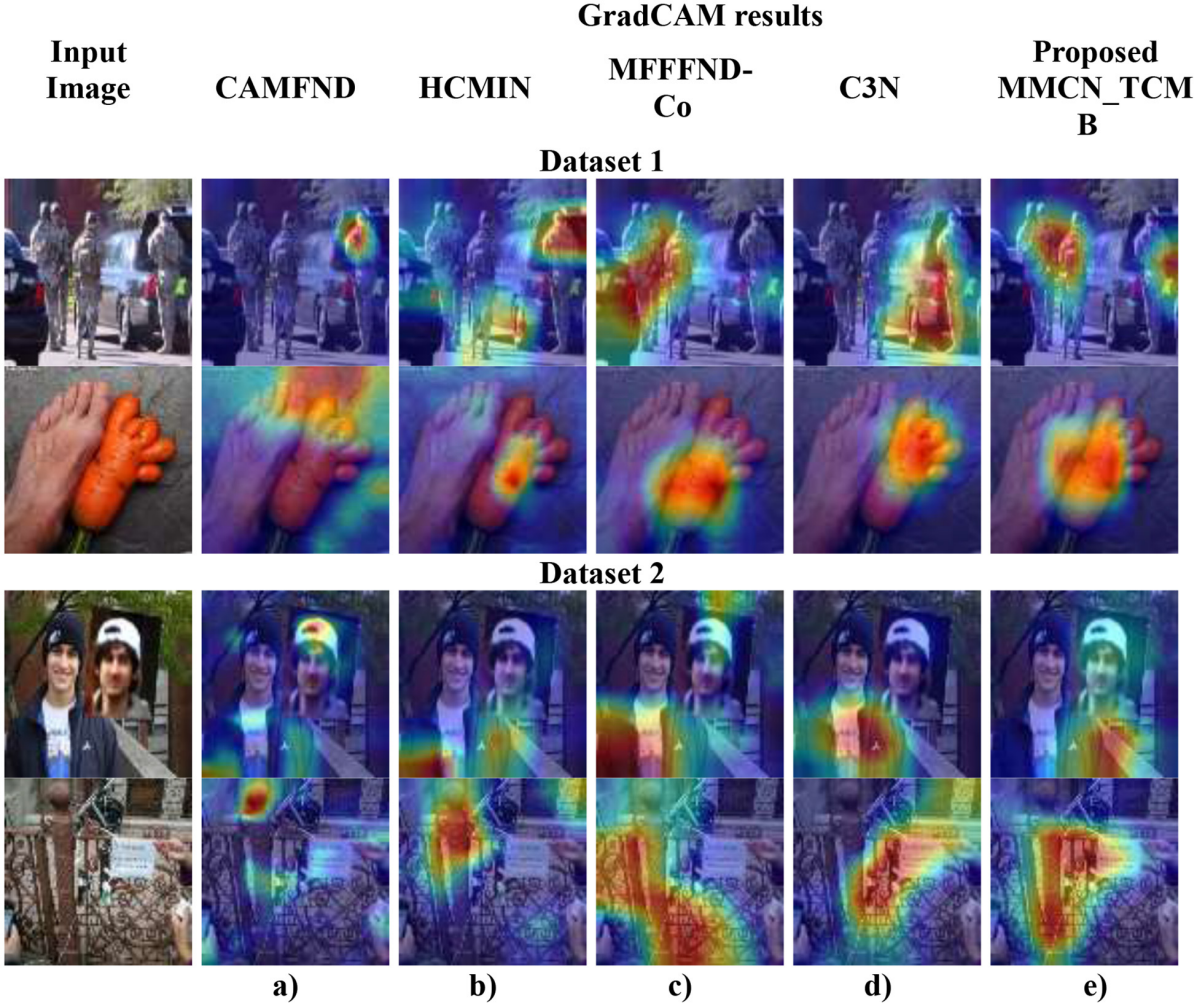
GradCAM assessment for dataset 1 and dataset 2 with **(a)** CAMFND method, **(b)** HCMIN, **(c)** MFFFND-Co, **(d)** C3N, and **(e)** proposed MMCN_TCMB.

Figure 3 depicts the explainability analysis of MMCN_TCMB exploiting Gradient-weighted Class Activation Mapping (GradCAM) visualizations. GradCAM highlights influential image areas by producing heatmaps based on gradients from the final convolutional layer. The interpretability of the proposed mode is assessed by contrasting it to techniques, such as CAMFND (Guo et al., 2025), HCMIN (Li et al., 2025), MFFFND-Co (Cao et al., 2025), and the C3N model (Qiao et al., 2025). Figure 3a displays the GradCAM outcomes for CAMFND; in Figure 3b, the GradCAM output for the HCMIN approach is exhibited; the GradCAM result for MFFFND-Co is depicted in Figures 3c, d presents the GradCAM outcomes for the C3N model; and the GradCAM result for the MMCN_TCMB is displayed in Figure 3e.

### 4.6 Image results

The experimental results obtained by the MMCN_TCMB for detecting multimodal fake news while recognizing both real and fake news for dataset 1 and dataset 2 are detailed here.

#### 4.6.1 When considering dataset 1

**Figure 4:**
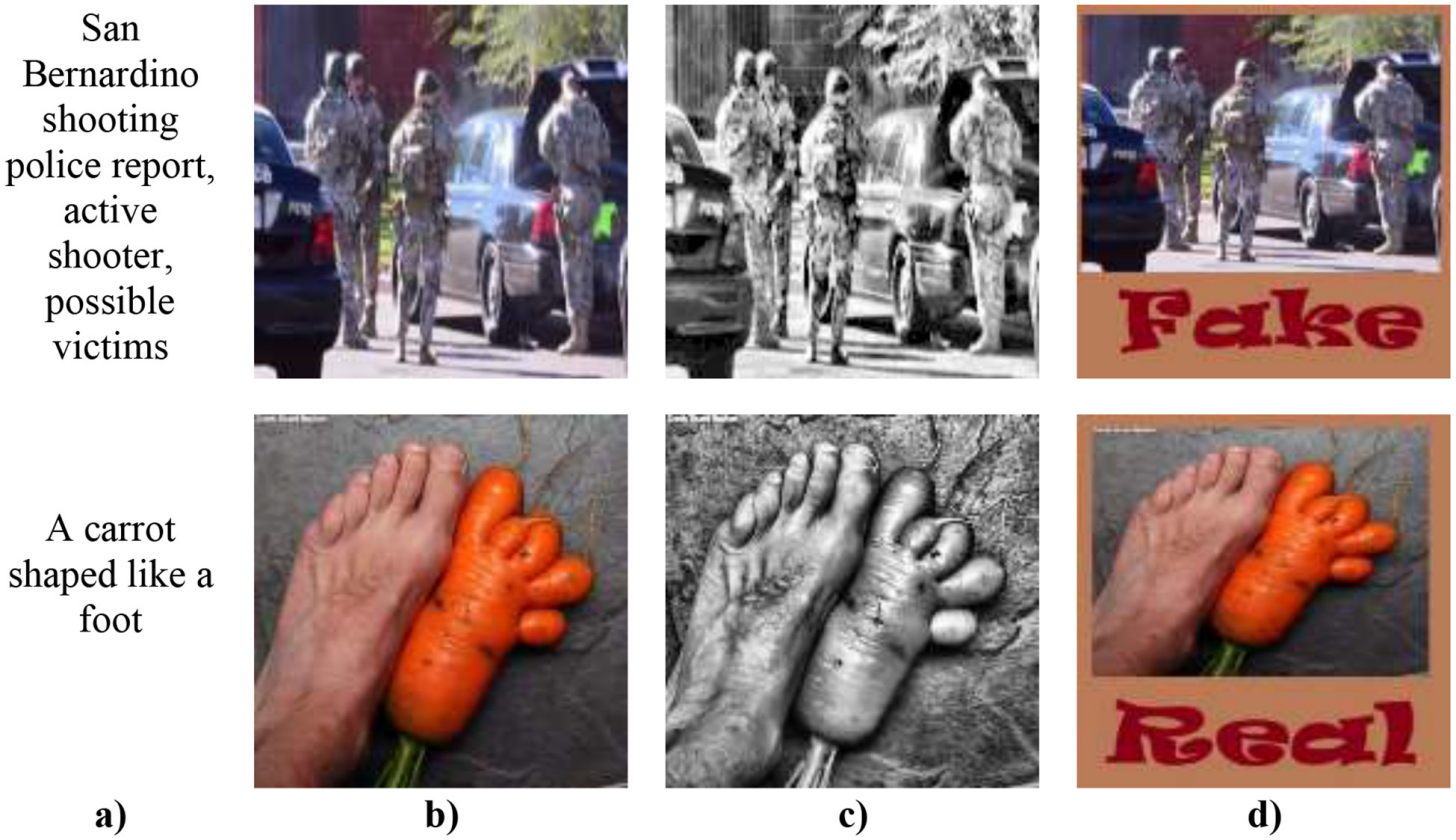
Experimental outcomes of MMCN_TCMB regarding database 1: **(a)** input text, **(b)** input image, **(c)** enhanced image, and **(d)** detected output.

The experimental results of the devised MMCN_TCMB for detecting multimodal fake news obtained when considering dataset 1 are epitomized in Figure 4. The input text is presented in Figure 4a; in Figure 4b, the input image is portrayed; Figure 4c displays an enhanced image; and detected output is depicted in Figure 4d.

#### 4.6.2 When considering database 2

**Figure 5:**
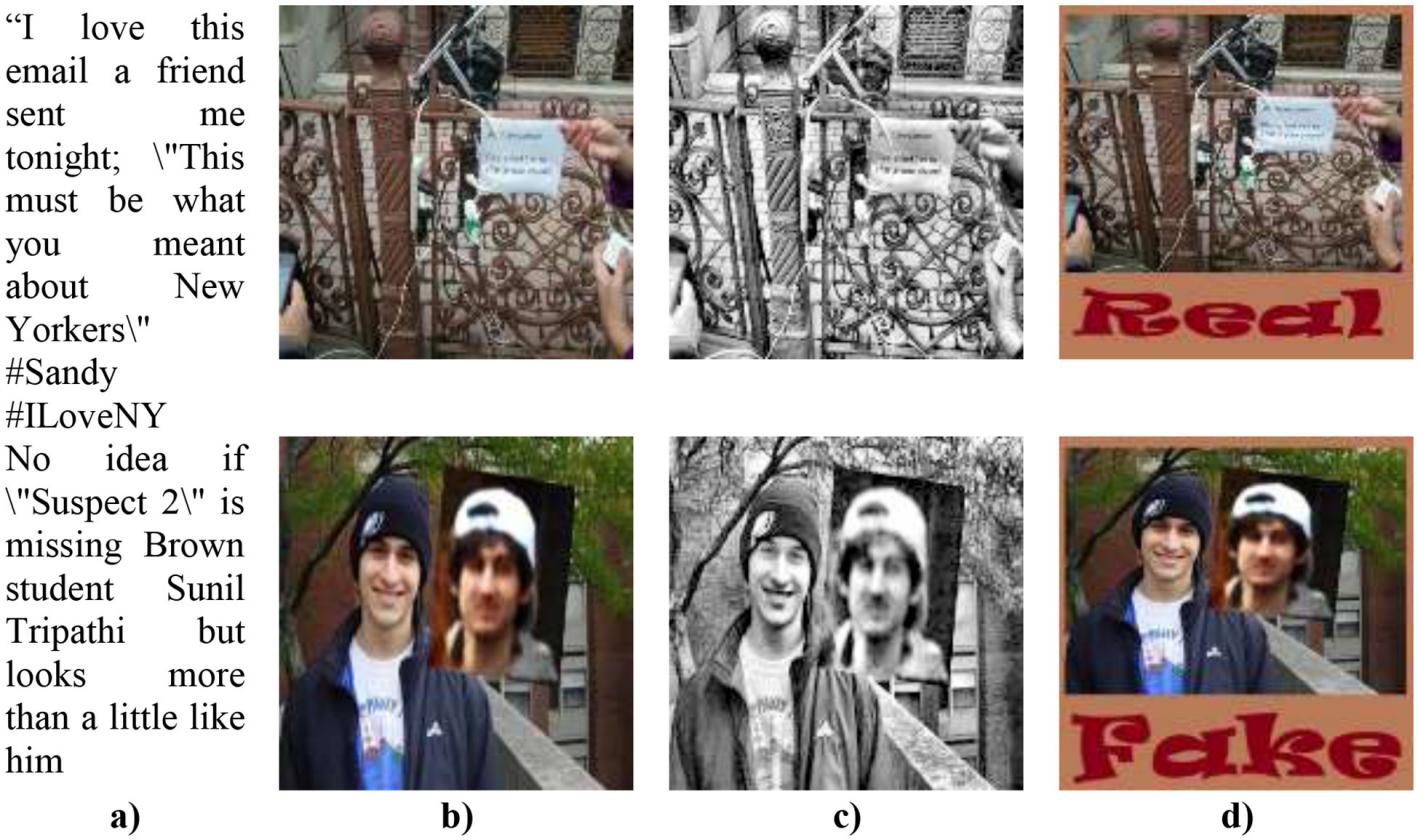
Image outcomes of MMCN_TCMB concerning dataset 2: **(a)** input text, **(b)** input image, **(c)** enhanced image, and **(d)** detected output.

The image outcomes obtained by the proposed MMCN_TCMB for detecting multimodal fake news when considering database 2 are given in Figure 5. In Figure 5a, the input text is presented, and the input image is portrayed in Figure 5b. Meanwhile, Figure 5c displays the enhanced image, and the detected output is depicted in Figure 5d.

### 4.7 Assessment for detecting fake news

Here, the ablation study, *K*-value assessment, and confusion matrix of the proposed MMCN_TCMB for detecting multimodal fake news based on setup 1 and setup 2 are revealed. In setup 1, dataset 1 is considered as the source domain and dataset 2 is regarded as the target domain. Similarly, in setup 2, dataset 2 is considered as the source domain and dataset 1 is taken as the target domain.

#### 4.7.1 Ablation assessment

The ablation assessment of the proposed MMCN_TCMB for fake news detection with regard to setup 1 and setup 2 is explicated in this section. Here, the ablation assessment is carried out by scenarios, such as MMCN+MBE (without sigmoid) and MMCN+Sigmoid (without MBE).

**(i) Ablation analysis for setup 1**

**Figure 6:**
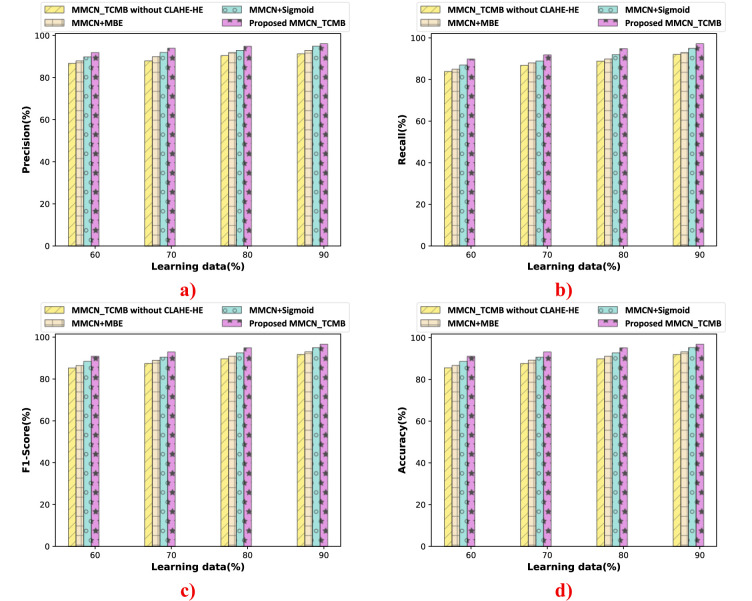
Ablation analysis of MMCN_TCMB for detecting multimodal fake news with setup 1: **(a)** precision, **(b)** recall, **(c)** F1-score, and **(d)** accuracy.

In Figure 6, the ablation analysis of MMCN_TCMB for detecting fake news based on setup 1 is depicted. The assessment of MMCN_TCMB regarding precision is exposed in Figure 6a. The precision obtained by MMCN_TCMB without CLAHE-HE is 86.788%, MMCN+MBE is 87.988%, MMCN+Sigmoid is 89.888%, and the developed MMCN_TCMB is 91.887%, when the learning data is 60%. Here, the precision of MMCN_TCMB is enhanced by 2.18% than MMCN+MBE. Figure 6b reveals the estimation of MMCN_TCMB with respect to recall. With learning data 70%, the recall provided by the MMCN_TCMB is 91.879%, and MMCN_TCMB without CLAHE-HE is 86.888%, MMCN+MBE is 87.988%, and MMCN+Sigmoid is 88.988%. The recall of MMCN_TCMB is higher by 3.22% than that of MMCN+Sigmoid. The analysis of MMCN_TCMB based on F1-score is represented in Figure 6c. The F1-score recorded by MMCN_TCMB without CLAHE-HE is 89.672%, MMCN+MBE is 90.877%, MMCN+Sigmoid is 92.486%, and the developed MMCN_TCMB is 94.882%, by exploiting 80% learning data. The F1-score of MMCN_TCMB is better by 2.70% when related to MMCN+Sigmoid. The analysis of MMCN_TCMB based on accuracy is represented in Figure 6d. The accuracy recorded by MMCN_TCMB without CLAHE-HE is 91.968%, MMCN+MBE is 93.247%, MMCN+Sigmoid is 95.247%, and the developed MMCN_TCMB is 96.924%, by exploiting 90% learning data.

**(ii) Ablation analysis for setup 2**

**Figure 7:**
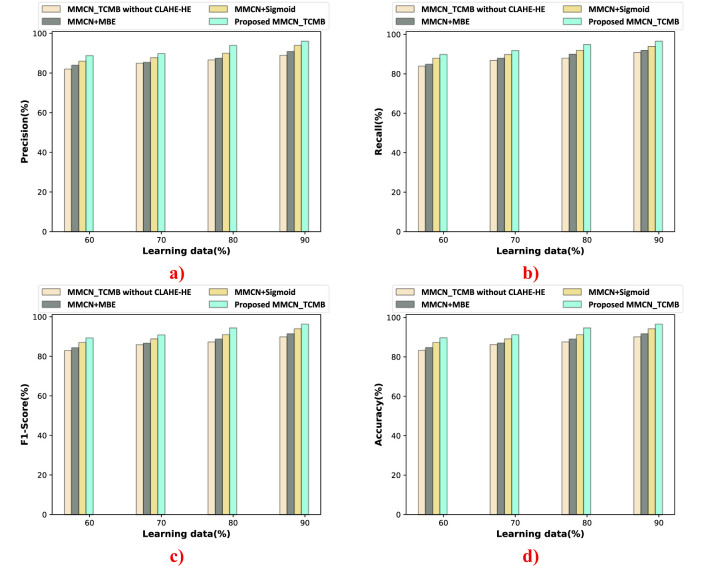
Ablation evaluation of MMCN_TCMB for detection of multimodal fake news regarding setup 2 with **(a)** precision, **(b)** recall, **(c)** F1-score, and **(d)** accuracy.

Figure 7 displays the valuation of MMCN_TCMB for detecting multimodal fake news concerning setup 2. In Figure 7a, the assessment of MMCN_TCMB concerning precision is revealed. The precision obtained by the proposed MMCN_TCMB is 90.887%, MMCN_TCMB without CLAHE-HE is 88.998%, MMCN+MBE is 93.988%, and MMCN+Sigmoid is 96.099%, with learning data of 90%. The precision of MMCN_TCMB is higher by 3.04% than that of MMCN+Sigmoid. Figure 7b displays the estimation of MMCN_TCMB concerning recall. By considering learning data of 60%, the recall gained by MMCN_TCMB without CLAHE-HE is 83.888%, MMCN+MBE is 84.888%, MMCN+Sigmoid is 87.988%, and the developed MMCN_TCMB is 89.877%. When compared to MMCN+Sigmoid, the recall of MMCN_TCMB is enhanced by 4.24%. In Figure 7c, the assessment of MMCN_TCMB regarding F1-score is displayed. When learning data is 70%, the F1-score acquired by the introduced MMCN_TCMB without CLAHE-HE is 85.929%, MMCN_TCMB is 90.866%, MMCN+MBE is 86.715%, and MMCN+Sigmoid is 88.815%. In comparison, the F1-score of MMCN_TCMB is enhanced by 3.65%, better than MMCN+MBE. The assessment of MMCN_TCMB regarding accuracy is displayed in Figure 7d. When learning data is 70%, the accuracy acquired by the introduced MMCN_TCMB without CLAHE-HE is 86.250%, MMCN_TCMB is 87.036%, MMCN+MBE is 89.136%, and MMCN+Sigmoid is 91.187%.

#### 4.7.2 K-value analysis

In this section, the *K*-value-based assessment of MMCN_TCMB for the detection of fake news using setup 1 and setup 2 is demonstrated. Here, CAMFND (Guo et al., 2025), HCMIN (Li et al., 2025), MFFFND-Co (Shu et al., 2018), C3N model (Qiao et al., 2025), MAGF (Shan et al., 2024), CM-MLF (He et al., 2025), and MFCL (Chen et al., 2025) are used to evaluate the effectiveness of the developed MMCN_TCMB for detecting fake news.

**(a)** ***K*****-value assessment for setup 1**

**Figure 8:**
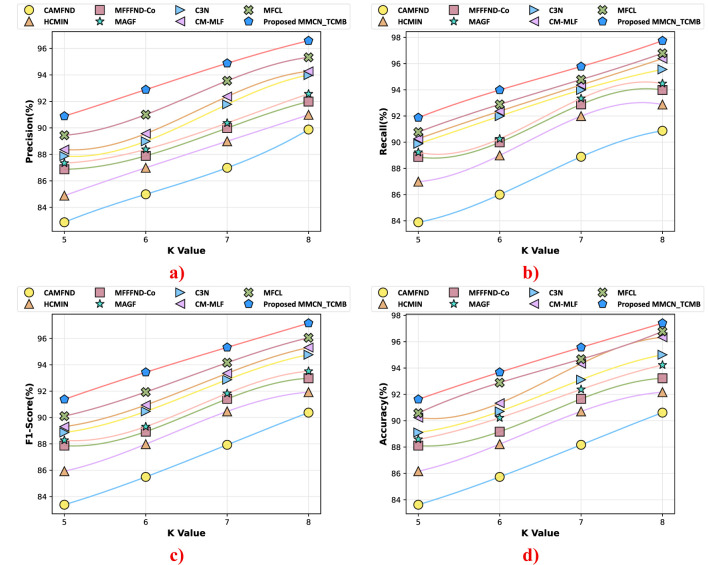
*K*-value assessment of MMCN_TCMB for multimodal fake news detection considering setup 1 by means of **(a)** precision, **(b)** recall, **(c)** F1-score, and **(d)** accuracy.

In Figure 8, the valuation of MMCN_TCMB for detecting fake news regarding setup 1 is elucidated. Figure 8a presents the evaluation of MMCN_TCMB based on precision. When the *K*-value is 5, the precision achieved by the proposed MMCN_TCMB is 90.887%, CAMFND is 82.877%, HCMIN is 84.887%, MFFFND-Co is 86.877%, MAGF is 87.339%, C3N is 87.877%, CM-MLF is 88.367%, and MFCL is 89.444%. Here, MMCN_TCMB outperforms the HCMIN by 4.20% in terms of precision. The evaluation of MMCN_TCMB based on recall is described in Figure 8b. The recall acquired by CAMFND is 85.999%, HCMIN is 88.990%, MFFFND-Co is 89.990%, MAGF is 90.257%, C3N is 91.988%, CM-MLF is 92.367%, MFCL is 92.888%, and the established MMCN_TCMB is 93.987%, with a *K*-value of 6. At this point, MMCN_TCMB attains a recall that is 2.24% higher than that of C3N. In Figure 8c, the evaluation of MMCN_TCMB concerning F1-score is revealed. With the *K*-value of 7, the F1-score measured by MMCN_TCMB is 95.325%, CAMFND is 87.928%, HCMIN is 90.464%, MFFFND-Co is 91.414%, MAGF is 91.829%, C3N is 92.869%, CM-MLF is 93.357%, and MFCL is 94.162%. The F1-score of MMCN_TCMB is improved over that of CAMFND by 3.18%. Figure 8d shows the evaluation of accuracy. With the *K*-value of 8, the accuracy measured by MMCN_TCMB is 97.402%, and for the existing approaches are as follows: CAMFND is 90.614%, HCMIN is 92.167%, MFFFND-Co is 93.221%, MAGF is 94.233%, C3N is 95.009%, CM-MLF is 96.333%, and MFCL is 96.777%.

**(b)** ***K*****-value assessment using setup 2**

**Figure 9:**
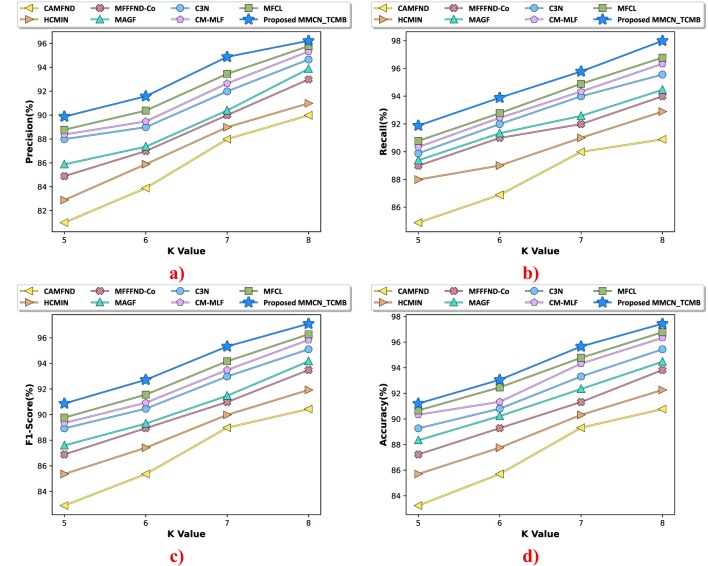
*K*-value assessment of MMCN_TCMB for detecting fake news using setup 2 with **(a)** precision, **(b)** recall, **(c)** F1-score, and **(d)** accuracy.

Figure 9 exposes the comparative valuation of MMCN_TCMB based on the learning set on setup 2. Figure 9a reveals the evaluation of MMCN_TCMB regarding precision. The precision measured by MMCN_TCMB is 96.223%, CAMFND is 89.988%, HCMIN is 90.988%, MFFFND-Co is 92.988%, MAGF is 93.877%, C3N is 94.657%, CM-MLF is 95.333%, and MFCL is 95.777%, when the *K*-value is 8. The precision of MMCN_TCMB is improved by 3.04% over that of MFFFND-Co. In Figure 9b, the analysis of MMCN_TCMB regarding recall is displayed. The recall measured by established MMCN_TCMB is 91.877%, and the value attained by the CAMFND is 84.888%, HCMIN is 87.989%, MFFFND-Co is 88.988%, MAGF is 89.378%, C3N is 89.887%, CM-MLF is 90.338%, and MFCL is 90.777%, by considering the *K*-value of 5. The recall of MMCN_TCMB is enhanced by 2.48% when compared to C3N. The valuation of MMCN_TCMB considering the F1-score is portrayed in Figure 9c. When the *K*-value is 6, the F1-score acquired by the CAMFND is 85.362%, HCMIN is 87.416%, MFFFND-Co is 88.943%, MAGF is 89.299%, C3N is 90.464%, CM-MLF is 90.928%, and MFCL is 91.562%, and the developed MMCN_TCMB is 92.719%. Here, the F1-score of MMCN_TCMB is 2.46% greater than that of CAMFN. The valuation of accuracy is portrayed in Figure 9d. When the *K*-value is 7, the accuracy acquired by the CAMFND is 89.315%, HCMIN is 90.320%, MFFFND-Co is 91.321%, MAGF is 92.340%, C3N is 93.316%, CM-MLF is 94.323%, MFCL is 94.777% and the developed MMCN_TCMB is 95.663%.

#### 4.7.3 Confusion matrix

The confusion matrix of MMCN_TCMB for detecting fake news for setup 1 and setup 2 is portrayed here.

**(i) Confusion matrix for setup 1**

**Figure 10:**
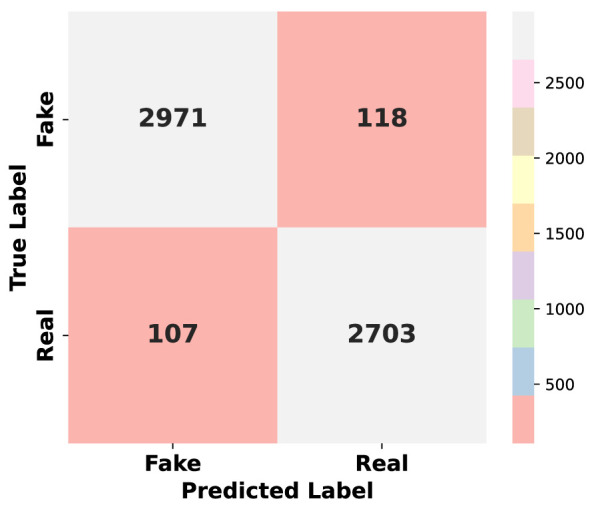
Confusion matrix for setup 1.

Figure 10 depicts the confusion matrix of MMCN_TCMB considering setup 1. The MMCN_TCMB achieves outstanding presentation in differentiating between real and fake news. It correctly classifies 2,703 real news instances and 2,971 fake news instances, while misclassifying only 118 fake news articles as real and 107 real news articles as fake.

**(ii) Confusion matrix for setup 2**

**Figure 11:**
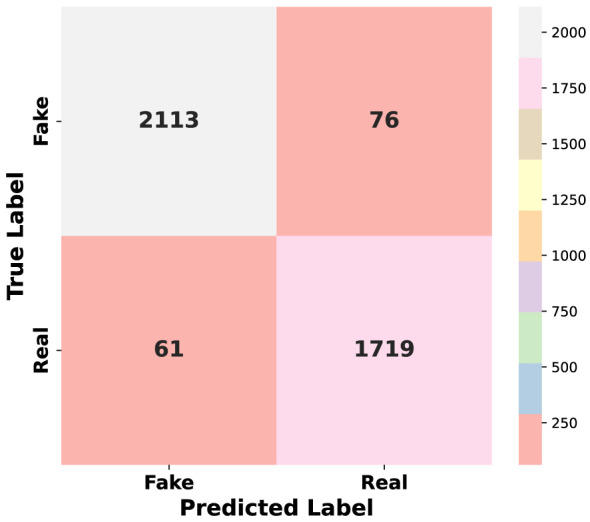
Confusion matrix for setup 2.

The confusion matrix of MMCN_TCMB for detecting multimodal fake news regarding setup 2 is depicted in Figure 11. MMCN_TCMB correctly classifies 2,113 fake and 1,719 real news data. Meanwhile, the MMCN_TCMB recorded 61 false negatives and 76 false positives.

#### 4.7.4 Receiver operating characteristics curve

**Figure 12:**
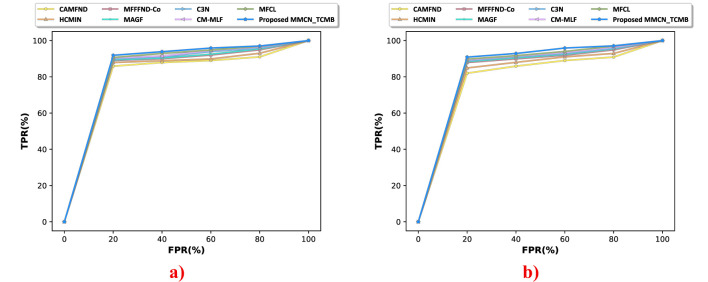
**(a, b)** ROC assessment for setup 1 and setup 2.

The ROC assessment of MMCN_TCMB for detecting multimodal fake news in terms of setup 1 and setup 2 is presented in Figure 12. In Figure 12a, the assessment of the ROC of MMCN_TCMB for detecting multimodal fake news regarding setup 1 is depicted. The TPR achieved by prevailing CAMFND is 88.989%, HCMIN is 89.879%, MFFFND-Co is 91.988%, MAGF is 92.467%, C3N is 93.877%, CM-MLF is 94.478%, MFCL is 94.770%, and the developed MMCN_TCMB is 95.877% with FPR of 60%. The analysis of the ROC of MMCN_TCMB for detecting multimodal fake news regarding setup 2 is portrayed in Figure 12b. By considering 80% of FPR, the TPR obtained by existing CAMFND is 90.877%, HCMIN is 92.887%, MFFFND-Co is 94.878%, MAGF is 95.378%, C3N is 95.766%, CM-MLF is 96.330%, MFCL is 96.888%, and the developed MMCN_TCMB is 97.100%.

### 4.8 Comparative discussion

Table 5 demonstrates the comparative discussion of MMCN_TCMB for detecting fake news based on metrics recorded by the MMCN_TCMB and the prevailing methods, namely CAMFND, HCMIN, MFFFND-Co, MAGF, C3N, CM-MLF, and MFCL, by utilizing a *K*-value of 8. The precision achieved by prevailing methods, such as CAMFND is 89.988%, HCMIN is 90.988%, MFFFND-Co is 92.988%, MAGF is 93.877%, C3N is 94.657%, CM-MLF is 95.333%, MFCL is 95.777%, and MMCN_TCMB is 96.223%. Meanwhile, the recall acquired by devised MMCN_TCMB is 97.988%, and the prevailing approaches, such as CAMFND is 90.888%, HCMIN is 92.887%, MFFFND-Co is 93.988%, MAGF is 94.455%, C3N is 95.555%, CM-MLF is 96.333%, and MFCL is 96.770%. Similarly, the F1-score measured by existing techniques, namely CAMFND is 90.436%, HCMIN is 91.928%, MFFFND-Co is 93.485%, MAGF is 94.165%, C3N is 95.104%, CM-MLF is 95.830%, MFCL is 96.271%, and the designed MMCN_TCMB is 97.098%. The highest accuracy obtained by the designed MMCN_TCMB is 97.436%.

**Table 5:** Comparative discussion.

Variations	Approaches							
	CAMFND	HCMIN	MFFFND-Co	MAGF	C3N	CM-MLF	MFCL	Proposed MMCN_TCMB
Setup 1								
Precision (%)	89.877	90.989	91.989	92.566	93.988	94.267	95.327	96.579
Recall (%)	90.870	92.877	93.988	94.477	95.555	96.378	96.788	97.746
F1 measure (%)	90.371	91.923	92.978	93.512	94.765	95.311	96.052	97.159
Accuracy (%)	90.614	92.167	93.221	94.233	95.009	96.333	96.777	97.402
Setup 2								
Precision (%)	89.988	90.988	92.988	93.877	94.657	95.333	95.777	96.223
Recall (%)	90.888	92.887	93.988	94.455	95.555	96.333	96.770	97.988
F1 measure (%)	90.436	91.928	93.485	94.165	95.104	95.830	96.271	97.098
Accuracy (%)	90.774	92.266	93.823	94.467	95.442	96.333	96.777	97.436

The large performance improvement of the proposed MMCN_TCMB compared to baseline models is mainly attributed to its effective multimodal learning and improved optimization strategy. The cross-attention mechanism enables efficient interaction between textual and visual features, allowing the model to capture complementary information that is not fully utilized in conventional fusion methods. In addition, the combination of BERT, Word2Vec, and TF-IGM provides rich semantic and statistical textual representations, while CLAHE-HE preprocessing enhances image quality for more discriminative ResNet feature extraction. Furthermore, the proposed TCMB loss function improves convergence and reduces learning bias, resulting in more accurate classification. These factors collectively contribute to the superior performance of the proposed model over the baseline methods.

### 4.9 Paired *t*-test

The statistical analysis of MMCN_TCMB for detecting multimodal fake news using the paired *t*-test is depicted in Table 6. The evaluation results demonstrate that the proposed model consistently surpassed CAMFND, HCMIN, MFFFND-Co, and C3N, across all key performance metrics, namely precision, accuracy, recall, and F1-score, with statistically significant improvements (*p* < 0.05), with established MMCN_TCMB attaining superior outputs with higher precision than CAMFND (*p* = 0.018, *t* = 2.777), HCMIN (*p* = 0.027, *t* = 2.369), MFFFND-Co (*p* = 0.032, *t* = 1.877), MAGF (*p* = 1.799, *t* = 0.035), C3N (*p* = 0.037, *t* = 1.578), CM-MLF (*p* = 1.379, *t* = 0.038), and MFCL (*p* = 1.119, *t* = 0.040) considering setup 1. Similarly, the devised MMCN_TCMB attained better recall than CAMFND (*p* = 0.014, *t* = 3.279), HCMIN (*p* = 0.019, *t* = 2.888), MFFFND-Co (*p* = 0.024, *t* = 2.590), MAGF (*p* = 2.380, *t* = 0.026), C3N (*p* = 0.030, *t* = 2.098), CM-MLF (*p* = 1.890, *t* = 0.032), and MFCL (*p* = 1.777, *t* = 0.034). Furthermore, the MMCN_TCMB figured a better F1-score than CAMFND (*p* = 0.022, *t* = 2.589), HCMIN (*p* = 0.032, *t* = 2.179), MFFFND-Co (*p* = 0.039, *t* = 1.590), MAGF (*p* = 1.489, *t* = 0.042), C3N (*p* = 0.044, *t* = 1.367), CM-MLF (*p* = 1.292, *t* = 0.045), and MFCL (*p* = 1.119, *t* = 0.047). Besides, the MMCN_TCMB figured a better accuracy than CAMFND (*p* = 3.010, *t* = 0.016), HCMIN (*p* = 2.589, *t* = 0.024), MFFFND-Co (*p* = 2.223, *t* = 0.027), MAGF (*p* = 2.009, *t* = 0.030), C3N (*p* = 1.789, *t* = 0.033), CM-MLF (*p* = 1.667, *t* = 0.036), and MFCL (*p* = 1.578, *t* = 0.038). A similar trend is observed in setup-2, in which the proposed MMCN_TCMB has the highest *t*-value and minimum *p*-value than the existing approaches.

**Table 6:** Paired *t*-test results of MMCN_TCMB.

Variations	Metrics	CAMFND	HCMIN	MFFFND-Co	MAGF	C3N	CM-MLF	MFCL
Proposed MMCN_TCMB								
Setup 1								
*T*-statistics	Precision (%)	2.777	2.369	1.877	1.799	1.578	1.379	1.119
	Recall (%)	3.279	2.888	2.590	2.380	2.098	1.890	1.777
	F1-score (%)	2.589	2.179	1.590	1.489	1.367	1.292	1.119
	Accuracy (%)	3.010	2.589	2.223	2.009	1.789	1.667	1.578
*p*-value	Precision (%)	0.018	0.027	0.032	0.035	0.037	0.038	0.040
	Recall (%)	0.014	0.019	0.024	0.026	0.030	0.032	0.034
	F1-score (%)	0.022	0.032	0.039	0.042	0.044	0.045	0.047
	Accuracy (%)	0.016	0.024	0.027	0.030	0.033	0.036	0.038
Setup 2								
*T*-statistics	Precision (%)	2.420	2.012	1.520	1.368	1.221	1.119	1.101
	Recall (%)	2.922	2.531	2.233	2.090	1.741	1.690	1.488
	F1-score (%)	2.232	1.822	1.233	1.120	1.010	1.010	1.004
	Accuracy (%)	2.768	2.337	2.010	1.867	1.666	1.356	1.256
*p*-value	Precision (%)	0.023	0.031	0.036	0.039	0.041	0.044	0.047
	Recall (%)	0.018	0.023	0.028	0.032	0.034	0.035	0.035
	F1-score (%)	0.026	0.036	0.044	0.046	0.048	0.049	0.049
	Accuracy (%)	0.020	0.021	0.025	0.029	0.031	0.032	0.032

## 5 Conclusion

Fake news is a growing issue in various fields, as it can misinform the public, deceive users, or negatively impact reputations. Still, various challenges such as bias in training data and high false positives and negatives affected the performance of the prevailing schemes. This study introduces an advanced model named MMCN_TCMB for detecting fake news from multimodal input. Initially, text and image data are gathered from Weibo and Twitter databases. Textual data are applied to BERT-based tokenization. Then, feature extraction is accomplished using Word2Vec and TF-IGM. Afterward, the image on a new post is considered, and the image is first pre-processed using CLAHE-HE hybrid denoising. Next, feature extraction is performed utilizing ResNet. Consequently, the extracted features from both text and images are merged and applied to MMCN_TCMB for detecting fake news. The learning rule of MMCN is upgraded utilizing the TCMB loss function. Additionally, the developed MMCN_TCMB achieved a better precision, recall, F1-score, and accuracy of 96.223, 97.988, 97.098, and 97.436%. In future work, the proposed framework will be extended to incorporate additional modalities, such as video and audio, along with text and images, to improve the robustness and applicability of fake news detection in real-world environments. Furthermore, lightweight and computationally efficient model architectures will be developed to reduce processing time and enable faster real-time fake news detection.

## Data Availability

The original contributions presented in the study are included in the article/supplementary material, further inquiries can be directed to the corresponding author.
